# Identification of biomarkers by machine learning classifiers to assist diagnose rheumatoid arthritis-associated interstitial lung disease

**DOI:** 10.1186/s13075-022-02800-2

**Published:** 2022-05-19

**Authors:** Yan Qin, Yanlin Wang, Fanxing Meng, Min Feng, Xiangcong Zhao, Chong Gao, Jing Luo

**Affiliations:** 1 Department of Rheumatology, Second Hospital of Shanxi Medical University, Taiyuan, 030001 Shanxi China; 2 The Shanxi Medical University, Taiyuan, 030001 Shanxi China; 3 Department of Pathology, Brigham and Women’s Hospital, Harvard Medical School, Boston, MA USA

**Keywords:** Interstitial lung disease, Rheumatoid arthritis, Krebs von den Lungen-6, D-dimer, Tumor markers, Machine learning algorithm

## Abstract

**Background:**

This study aimed to search for blood biomarkers among the profiles of patients with RA-ILD by using machine learning classifiers and probe correlations between the markers and the characteristics of RA-ILD.

**Methods:**

A total of 153 RA patients were enrolled, including 75 RA-ILD and 78 RA-non-ILD. Routine laboratory data, the levels of tumor markers and autoantibodies, and clinical manifestations were recorded. Univariate analysis, least absolute shrinkage and selection operator (LASSO), random forest (RF), and partial least square (PLS) were performed, and the receiver operating characteristic (ROC) curves were plotted.

**Results:**

Univariate analysis showed that, compared to RA-non-ILD, patients with RA-ILD were older (*p* < 0.001), had higher white blood cell (*p* = 0.003) and neutrophil counts (*p* = 0.017), had higher erythrocyte sedimentation rate (*p* = 0.003) and C-reactive protein (*p* = 0.003), had higher levels of KL-6 (*p* < 0.001), D-dimer (*p* < 0.001), fibrinogen (*p* < 0.001), fibrinogen degradation products (*p* < 0.001), lactate dehydrogenase (*p* < 0.001), hydroxybutyrate dehydrogenase (*p* < 0.001), carbohydrate antigen (CA) 19–9 (*p* < 0.001), carcinoembryonic antigen (*p* = 0.001), and CA242 (*p* < 0.001), but a significantly lower albumin level (*p* = 0.003). The areas under the curves (AUCs) of the LASSO, RF, and PLS models attained 0.95 in terms of differentiating patients with RA-ILD from those without. When data from the univariate analysis and the top 10 indicators of the three machine learning models were combined, the most discriminatory markers were age and the KL-6, D-dimer, and CA19-9, with AUCs of 0.814 [95% confidence interval (CI) 0.731–0.880], 0.749 (95% CI 0.660–0.824), 0.749 (95% CI 0.660–0.824), and 0.727 (95% CI 0.637–0.805), respectively. When all four markers were combined, the AUC reached 0.928 (95% CI 0.865–0.968). Notably, neither the KL-6 nor the CA19-9 level correlated with disease activity in RA-ILD group.

**Conclusions:**

The levels of KL-6, D-dimer, and tumor markers greatly aided RA-ILD identification. Machine learning algorithms combined with traditional biostatistical analysis can diagnose patients with RA-ILD and identify biomarkers potentially associated with the disease.

**Supplementary Information:**

The online version contains supplementary material available at 10.1186/s13075-022-02800-2.

## Introduction

Rheumatoid arthritis (RA) is a common systemic inflammatory disease caused by the interactions between genetic and environmental factors; the prevalence in the general population ranges from 0.5 to 2%. RA is characterized by synovitis and erosive destruction of the cartilage and bone [1, 2]. Notably, various extra-articular manifestations are common [3]. Pulmonary involvement is particularly common, potentially affecting all compartments of the respiratory system, including the serosal, airway, and/or parenchymal tissues [4]. Interstitial lung disease (ILD) caused by lung parenchymal damage is often the most devastating lung issue; the prevalence ranges from 6 to 30%. ILD is one of the leading causes of morbidity and premature mortality in RA patients [3, 5]. RA-ILD was first reported by Ellman and Ball in 1948 [6]. In a recent study, the 1- and 5-year mortality rates were 13.9 and 39.0%, respectively, compared to 3.8 and 18.2% in RA patients without ILD [7]. Hence, early recognition and monitoring of RA-ILD is paramount to potentially alter the disease course.

RA-ILD diagnosis requires multidisciplinary discussion and evaluation of patient’s medical history, clinical characteristics, laboratory indicators, high-resolution computed tomography (HRCT), pulmonary function test (PFT), and even lung biopsy [8]. Although ILD is well-recognized as a common comorbidity of RA, the present assessment tools (chest X-ray, HRCT, and PFT) may not be optimal for all patients. Radiation exposure and high cost may limit the use of HRCT in clinical practice, especially in younger patients and those for whom disease progression must be monitored over time [9]. Therefore, biomarkers assisting RA-ILD diagnosis, and that aid prognosis, assessment, and follow-up are urgently required.

Krebs von den Lungen-6 (KL-6) is a mucin-like, high-molecular-weight glycoprotein expressed on the surface membranes of alveolar and bronchiolar epithelial cells, particularly on type II pneumocytes that are damaged or regenerating; KL-6 is then secreted into the bloodstream through damaged alveolar basement membrane [10]. Recent study demonstrated that KL-6 plays important roles in the diagnosis, prognostic assessment, and risk stratification of connective tissue disease-related interstitial lung disease (CTD-ILD) [11]. Additionally, the development of tumor markers may also contribute to ILD; their diagnostic utilities have been investigated. The levels of carbohydrate antigen (CA) 19–9, CA125, CEA, and CA15-3 were increased compared to a control group of RA-non-ILD patients [12, 13]. D-dimer is the end-product of cross-linked fibrinolysis and is involved in the acute phase of inflammation; it may thus contribute to the pathophysiology of RA-ILD [14]. Tian et al. [15] assessed the levels of various serum markers in a cohort of CTD-ILD patients and found that the D-dimer levels were elevated. Based on this, we hypothesized that integration of these indicators might aid the screening of RA patients with ILD. However, few integrated models that effectively differentiate RA patients with and without ILD have been reported. Thus, an integrated model that combines multiple biomarkers to diagnose RA-ILD is pressing.

Over the past decade, great strides have been made in machine learning (a branch of artificial intelligence). Computers simulate human learning, build analytical models as they learn by example, train and evaluate models, and self-improve over multiple cycles in terms of their predictive powers. Machine learning allows researchers to use complex data and develop self-trained strategies to predict the characteristics of new samples. The algorithms have found applications in clinical fields, including disease prediction, diagnosis, and prognosis, and in drug discovery [16–18]. A method that combines multiple biomarkers to diagnose RA-ILD would be optimal. Here, we used machine learning to integrate data on the levels of KL-6, tumor biomarkers, and routine laboratory parameters and clinical features in order to identify the biomarkers that best diagnose RA-ILD.

## Materials and methods

### Patients

This was a retrospective analysis of 153 patients (57 new-onset RA patients and 96 treated RA patients hospitalized due to disease relapse, 103 females and 50 males, mean age 53.82 ± 14.29 years) who met the the definitive 1987 RA classification criteria of the American College of Rheumatology (ACR) at the Second Hospital of Shanxi Medical University between February 2020 and November 2021 [19]. All patients were divided into two groups: the RA-ILD group and the RA-non-ILD group. ILD was diagnosed by a rheumatologist and radiologist based on HRCT-revealed reticular abnormalities and honeycombing and clinical features. The disease activity was evaluated using the disease activity score 28-ESR [DAS28(ESR)], which is the most frequently used clinical tool to determine RA disease severity [20]. Patients who were younger than 18 years of age or pregnant, or who suffered from a malignant disease (a cancer/tumor), sarcoidosis, amyloidosis, an infection (bacteria, viral, or fungal), or other autoimmune diseases, were excluded. All patients had stopped drug treatment for more than 3 months at the time of sampling. The study was approved by the ethics committee of the Second Hospital of Shanxi Medical University (2016KY007). Informed consent was obtained from all individuals.

### Clinical and laboratory indices

The clinical parameters of all patients were retrospectively collected; these included age, gender, disease duration, and clinical manifestations (the tender joint count [TJC], swollen joint count [SJC], and DAS28). The routine laboratory data included the white blood cell (WBC), red blood cell (RBC) count, hemoglobin (Hb), platelet (PLT), lymphocyte (LYMPH), and neutrophil (NEUT); erythrocyte sedimentation rate (ESR), C-reactive protein (CRP), and immune globulin (Ig) G, IgM and IgA; alanine transaminase (ALT), aspartate aminotransferase (AST), serum total protein (TP), albumin (ALB), globulin (GLO), lactate dehydrogenase (LDH), and lactate dehydrogenase (HBDH); and RA-related autoantibodies (rheumatoid factor [RF], anti-nuclear antibodies [ANA], anti-perinuclear factor [APF], anti-keratin antibodies [AKA], anti-cyclic citrullinated peptide antibody [CCP], and anti-mutated citrulline vimentin [MCV]). We also recorded the levels of D-dimer, fibrinogen degradation products (FDP), fibrinogen (FIB), and tumor markers (CA19-9, CA125, CA153, CA242, neuron-specific enolase [NSE], carcinoembryonic antigen [CEA], squamous cell carcinoma antigen [SCC], and alpha-fetoprotein [AFP]).

### KL-6 assay

Peripheral venous blood samples from RA patients were collected immediately after admission and before drug administration (within 24 h of hospitalization) and stored at –80 °C. The levels of KL-6 were measured using the Kaeser 6600 chemiluminescent immunoassay following the manufacturer’s instructions.

### Statistical analysis

**Fig. 1:**
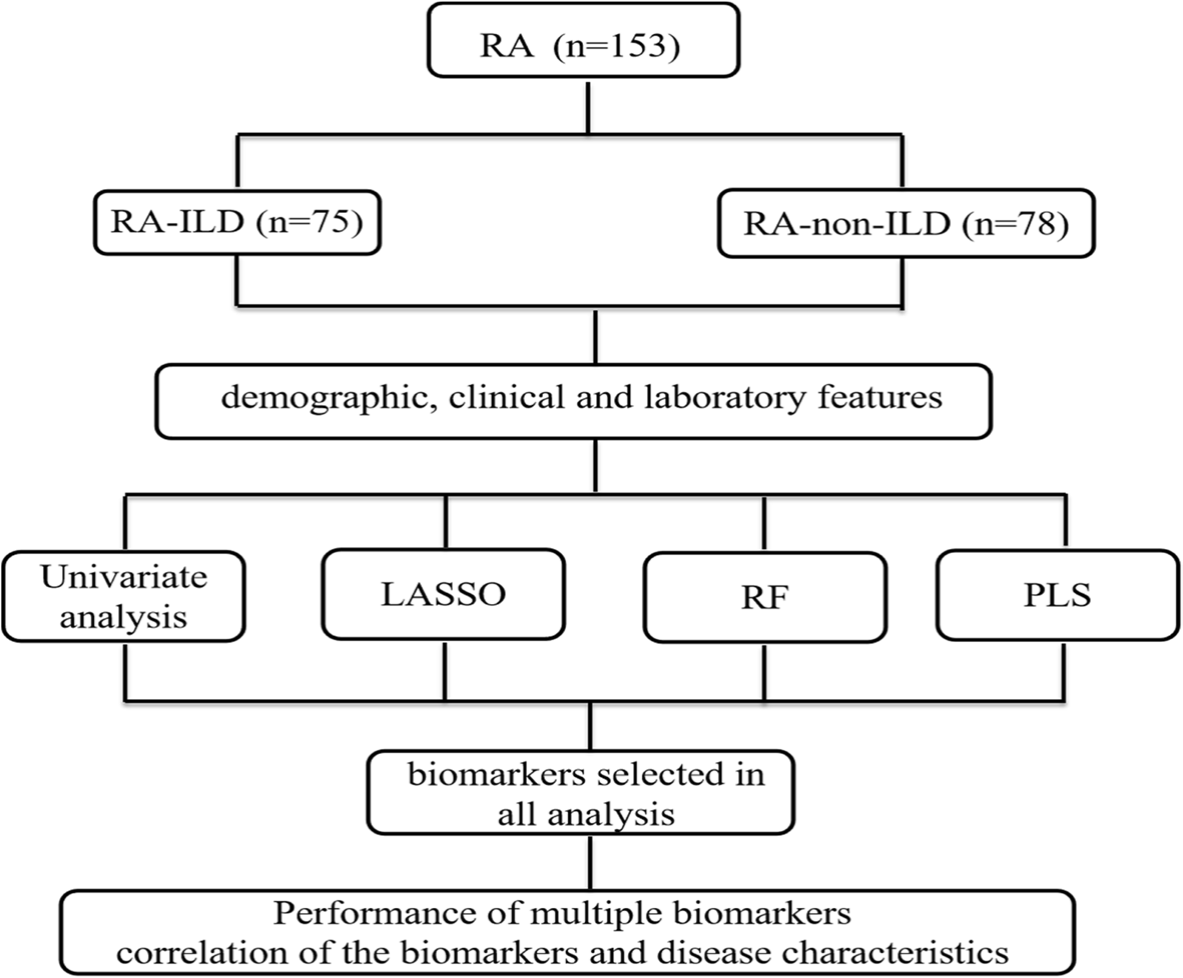
The design and analysis plan flow diagram in this study. RA, rheumatoid arthritis; ILD, interstitial lung disease; LASSO, least absolute shrinkage and selection operator; RF, random forest; PLS, partial least square

All data were analyzed using the SPSS 22.0, R package (version 4.0.2) and MedCalc software. In univariate analysis, the data were described as mean ± SD or as median (Q25, Q75) for continuous variables, and were compared using the independent samples t-test or the Mann–Whitney U test, respectively. The effect of age on various parameters was corrected with the aid of the covariance test. The chi-square test was employed to compare categorical variables expressed as numbers with percentages. Next, a total of 34 continuous variables described in the univariate analysis were incorporated into the least absolute shrinkage and selection operator (LASSO), random forest (RF), and partial least square (PLS) and were employed to classify patients with RA-ILD and RA-non-ILD. In this study, machine learning was trained on 70% subsets with tenfold cross-validation; the 30% holdout subsets were used for validation of the final model. We set 10 random seeds, and each seed corresponded to tenfold cross-verification; we got 10 different data segmentation “optimal model” by re-iterating tenfold cross-validation. We obtained the ranking of important variables of each “optimal model” through varlmp function (Package caret version 6.0). The top 10 most-weighted features were designated as an important feature when the AUC of LASSO, RF, and PLS was biggest in the 10 “optimal model,” respectively. Overall important biomarkers were selected on the basis of being simultaneously important of three machine learning algorithms and had significant differences in univariate analysis. The performance of biomarkers was evaluated by drawing receiver operating characteristic (ROC) curves. The area under curve (AUC), the cut-off, sensitivity, specificity, positive likelihood ratio (+ LR), negative likelihood ratio (-LR), Youden index, and comparisons of these biomarkers were performed by MedCalc software. Spearman rank correlation analysis was used to analyze correlations between biomarkers and disease activity. Figure 1 shows the study design and the analytical plan flow. The *p* value < 0.05 was considered to indicate statistical significance.

## Results

### Demographic and clinical characteristics of RA patients

The 153 RA patients were divided into RA-ILD group (*n* = 75) and RA-non-ILD (*n* = 78). Before employing the machine learning algorithms, we used a conventional biostatistics approach to analyze the differences between RA-ILD (45 females, 30 males) and RA-non-ILD (58 females, 20 males) patients. The details of demographic, clinical, and laboratory features between the two groups were summarized in Table 1. The a higher frequency of RA-ILD than RA-non-ILD in men, but no significant differences (*p* = 0.058). There was no significant differences in smoking history (*p* = 0.101) between the RA-ILD and RA-non-ILD groups. However, the RA-ILD patients were significantly older in than RA-non-ILD patients (62.84 ± 8.71 vs. 45.15 ± 13.31 years, *p* < 0.001). The clinical manifestations such as TJC and SJC were similar in the two groups (both *p* > 0.05). Compared to RA-non-ILD patients, the patients with RA-ILD exhibited a higher WBC count (*p* = 0.003), NEUT count (*p* = 0.017), ESR (*p* = 0.003), and CRP (*p* = 0.003), but a significantly lower ALB level (*p* = 0.003).

**Table 1:** Comparisons of the demographic, clinical, and laboratory features between the RA-ILD and the RA-non-ILD group

	RA-ILD (*n* = 75)	RA-non-ILD (*n* = 78)	*P*
Demographic parameters			
Age (years)	62.84 ± 8.71	45.15 ± 13.31	** < 0.001**
Female/male, *n*	45/30	58/20	0.058
Smoker, *n* (%)	16 (21.33)	9 (11.54)	0.101
Clinical parameters			
TJC	8.00 (2.00, 23.00)	5.00 (2.00, 14.25)	0.177
SJC	2.00 (0.00, 8.00)	2.00 (0.00, 8.00)	0.338
DAS28 (ESR)	5.59 (4.01, 6.56)	5.01 (3.50, 6.25)	0.162
Disease duration (years)	5 (0.75, 16.00)	3.00 (1.00, 10.00)	0.280
Laboratory parameters			
WBC (*10^9^/L)	7.36 (6.11, 8.47)	6.16 (5.26, 7.75)	**0.003**
RBC (*10^12^/L)	4.25 ± 0.47	4.25 ± 0.47	0.970
Hb (g/L)	123.39 ± 16.18	121.00 ± 16.88	0.373
PLT (*10^9^/L)	248.00 (195.00, 330.00)	294.00 (221.5, 347.25)	0.154
LYMPH (*10^9^/L)	1.68 (1.32, 2.38)	1.57 (1.28, 1.85)	0.117
NEUT (*10^9^/L)	4.77 (3.76, 6.17)	3.88 (3.29, 5.40)	**0.017**
ALT (U/L)	14.60 (11.13, 18.23)	13.90 (9.75, 21.45)	0.677
AST (U/L)	17.35 (14.50, 21.80)	16.80 (13.40, 20.00)	0.125
TP (g/L)	67.78 ± 7.41	68.55 ± 6.00	0.485
ALB (g/L)	35.61 ± 4.80	38.11 ± 4.88	**0.002**
GLO (g/L)	32.17 ± 7.01	30.51 ± 5.57	0.111
ESR (mm/h)	57.00 (30.00, 95.00)	36.00 (18.00, 67.00)	**0.003**
CRP (mg/L)	26.00 (9.04, 69.13)	11.70 (3.08, 38.00)	**0.003**
IgG (g/L)	12.75 (10.10, 15.93)	12.30 (11.03, 15.40)	0.852
IgA (g/L)	3.34 ± 1.22	3.12 ± 1.20	0.307
IgM (g/L)	1.42 (0.98, 1.82)	1.33 (0.99, 2.03)	0.574
RF ( +), *n* (%)	63 (84.00)	56 (71.79)	0.069
Anti-CCP ( +), *n* (%)	59 (78.67)	53 (67.95)	0.135

All data are reported as the numbers, mean ± SD or medians (IQR). The categorical variables are compared by chi-squared, Mann–Whitney *U* test, or independent sample *T* test and were used for continuous variables

*TJC*, tender joint count; *SJC*, swollen joint count; *WBC*, white blood cell; *RBC*, red blood cell; *Hb*, hemoglobin; *PLT*, platelet count; *LYMPH*, lymphocyte; *NEUT*, neutrophile; *ALT*, alanine transaminase; *AST*, aspartate aminotransferase; *TP*, serum total protein; *ALB*, albumin; *GLO*, globulin; *CRP*, C-reactive protein; *Ig*, immune globulin

### KL-6 and tumor markers were increased in patients with RA-ILD  

**Fig. 2:**
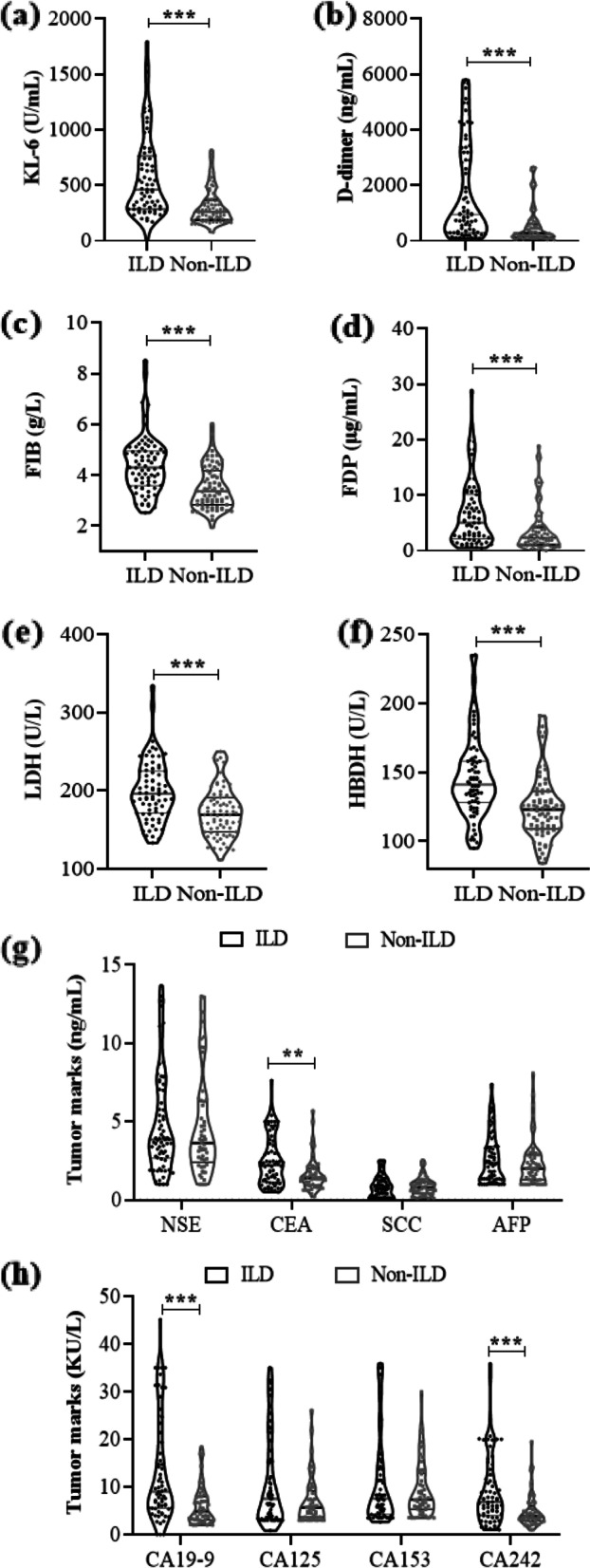
Elevated biomarkers level in RA-ILD patients. The levels of KL-6 (**a**), D-dimer (**b**), FIB (**c**), FDP (**d**), LDH (**e**), HBDH (**f**), CEA (**g**),CA19-9, and CA153 (**h**) were significantly higher in RA-ILD patients. ILD, rheumatoid arthritis-related interstitial lung disease; Non-ILD, rheumatoid arthritis-without interstitial lung disease; KL-6, Krebs von den Lungen-6; FIB, fibrinogen; FDP, fibrinogen degradation products; LDH, lactate dehydrogenase; HBDH, hydroxybutyrate dehydrogenase; NSE, neuron-specific enolase; CEA, carcinoembryonic antigen; SCC, squamous cell carcinoma antigen; AFP, alpha-fetoprotein; CA, carbohydrate antigen

The KL-6 level was significantly higher in the RA-ILD than the RA-non-ILD group [470.46 (288.92, 804.88) U/mL vs. 260.77 (188.07, 368.79) U/mL, *p* < 0.001]. The levels of CEA [2.30 (1.21, 3.81) ng/mL vs. 1.39 (0.95, 2.03) ng/mL, *p* = 0.001], CA19-9 [9.14 (5.59, 22.44) KU/L vs. 5.04 (3.12, 8.01) KU/L, *p* < 0.001] and CA242 [6.89 (4.01, 13.14) KU/L vs. 3.85 (2.86, 6.01) KU/L, *p* < 0.001] were higher in patients with RA-ILD than RA-non-ILD, but no significant between-group difference was noted for NSE, SCC, AFP, CA125, and CA153 (all *p* > 0.05). Meanwhile, the levels of D-dimer [961.50 (294.50, 3360.25) ng/mL vs. 263.00 (138.00, 604.00) ng/mL, *p* < 0.001], FIB [4.30 (3.59, 4.95) g/L vs. 3.37 (2.83, 4.18) g/L, *p* < 0.001], FDP [5.40 (2.31, 10.61) μg/mL vs. 2.39 (1.07, 4.43) μg/mL, *p* < 0.001)], LDH [197.00 (171.75, 226.50) U/L vs. 170.00 (148.00, 191.75) U/L, *p* < 0.001] and HBDH [142.50 (128.00, 159.25) U/L vs. 123.50 (109.00, 136.75) U/L, *p* < 0.001] in patients with RA-ILD were significantly higher than in those with RA-non-ILD (Fig. 2). Thus, results suggested that these parameters could be potentially promising biomarkers of RA-ILD.

### Multiple machine learning models distinguishing RA-ILD from RA

**Fig. 3:**
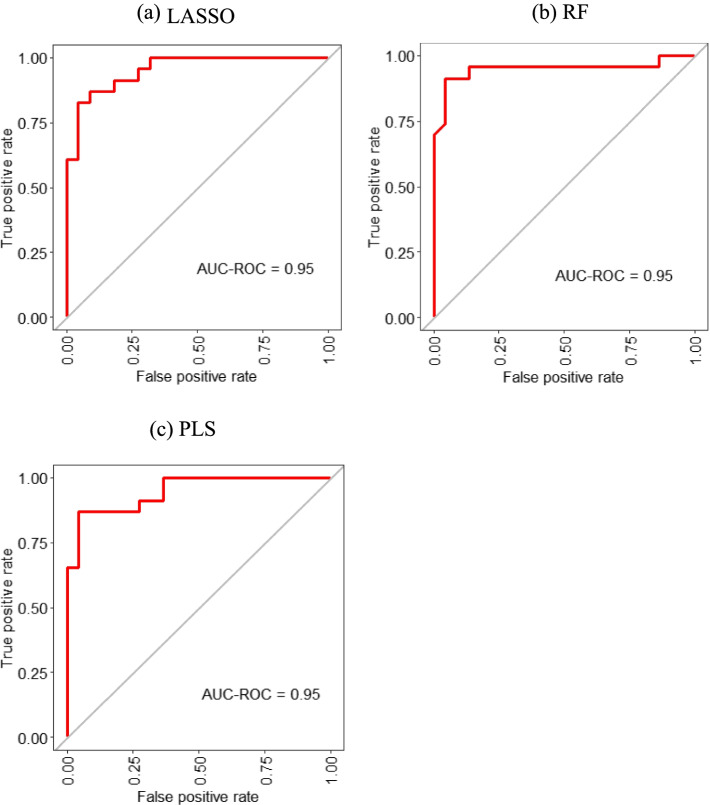
Machine learning approaches are effective at separating RA-ILD and RA-non-ILD subjects. The maximum of area under the ROC curve of LASSO (**a**), RF (**b**, **c**)

**Fig. 4:**
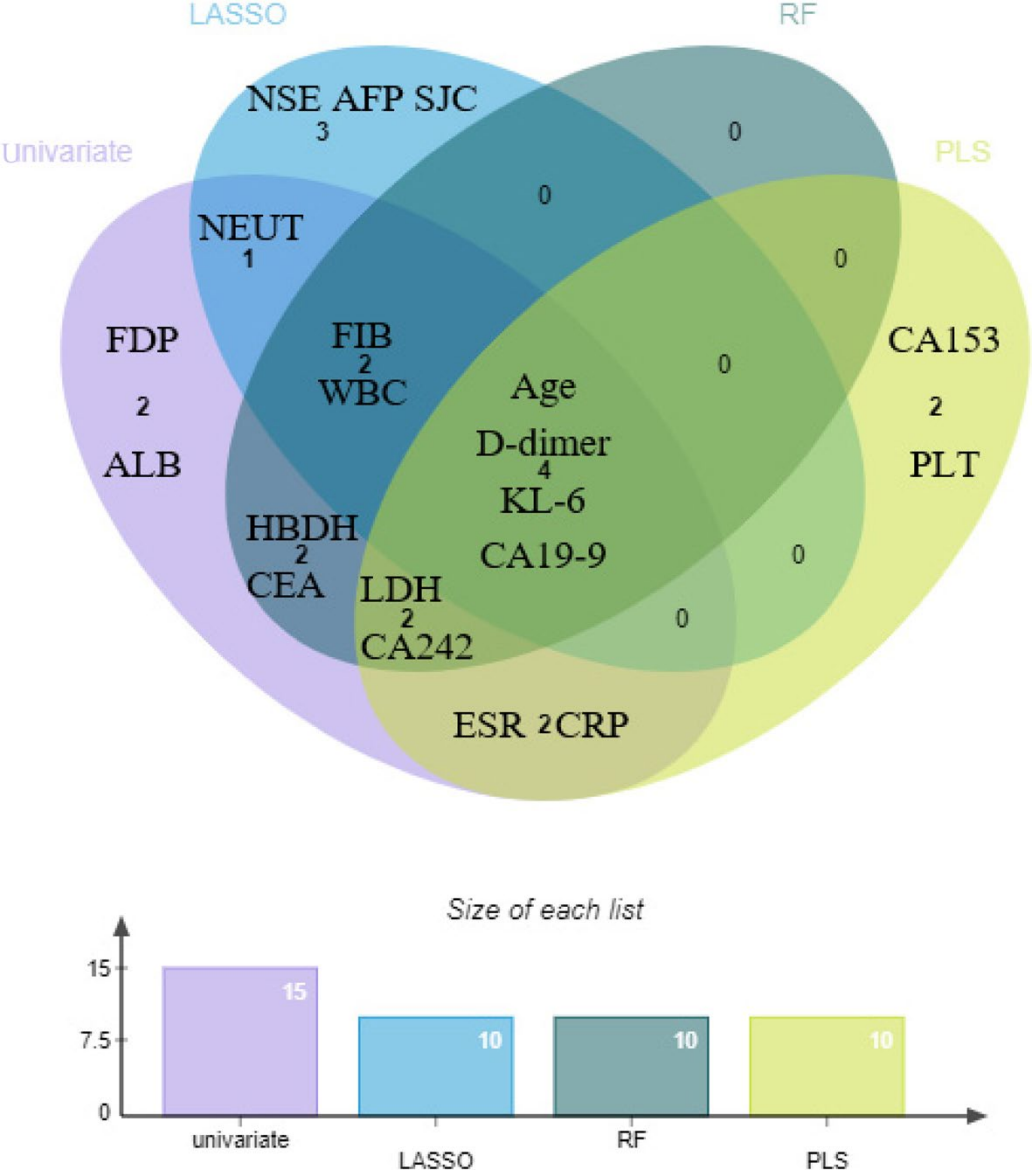
Venn diagram showing the four characteristic markers identified by the univariate analysis, LASSO, RF, and PLS model

We used the LASSO, RF, and PLS to further distinguish RA-ILD and RA-non-ILD patients and to screen for valuable variables. The classification accuracy of models remained stable in 10 runs; the AUCs of LASSO, RF, and PLS were 0.84 to 0.95, 0.85 to 0.95, and 0.81 to 0.95, respectively (Supplemental Table 1). ROC analysis revealed a max AUC of 0·95 (accuracy 95%), indicating outstanding efficiency in discriminating between RA-ILD from RA-non-ILD patients (Fig. 3). The top 10 contributing features were age, KL-6, FIB, D-dimer, CA199, WBC, NEUT, NSE, AFP, and SJC for LASSO; age, KL-6, FIB, D-dimer, CA199, CA242, LDH, CEA, HBDH, and WBC count for RF; and age, KL-6, D-dimer, CA19-9, CA242, LDH, CRP, ESR, CA153, and PLT for PLS (Fig. 4).

### Clinical values of biomarkers in diagnosing ILD in RA patients

**Fig. 5:**
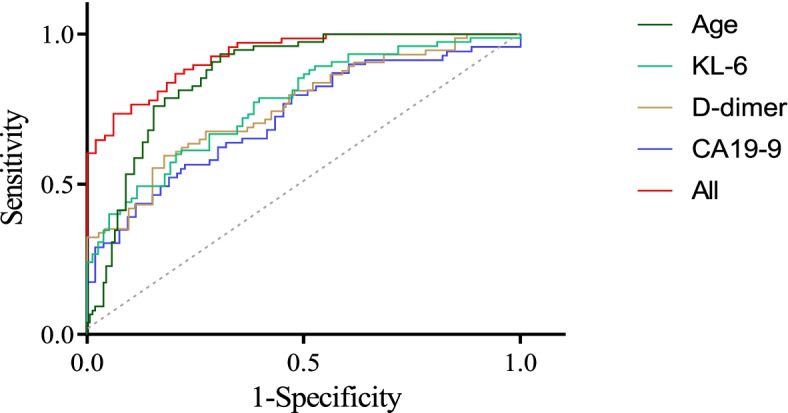
Important biomarkers were selected from multiple analyses and ROC curves were plotted. The ROCs of age, KL-6, D-dimer, and CA19-9, and their combination were plotted to differentiate RA-ILD from RA-non-ILD. The ROC curve for the combination of age, KL-6, D-dimer, and CA19-9 exhibited an AUC of 0.928

Based on the LASSO, RF, and PLS, and univariate analysis, four simultaneously important indicators were identified: age, KL-6, D-dimer, and CA19-9. The ROC curves of these four indicators were plotted in Fig. 5. ROC curve analysis revealed that the AUC of age was 0.814 (95% CI 0.731–0.880, *p* < 0.001), with a sensitivity of 93.33% and a specificity of 67.95%. The cut-off value for KL-6 was set at 373.65 U/mL, with a sensitivity of 61.33% and a specificity of 78.21% [AUC 0.749 (95% CI 0.660–0.824), *p* < 0.001]. The AUCs for D-dimer and CA19-9 were 0.749 (95% CI 0.660–0.824, *p* < 0.001) and 0.727 (95% CI 0.637–0.805, *p* < 0.001), respectively. Furthermore, the ROC curve for the combination of age, KL-6, D-dimer, and CA19-9 exhibited an AUC of 0.928 (95% CI 0.865–0.968, *p* < 0.001) with a sensitivity of 83.82% and a specificity of 81.63%. The AUC provided by the biomarker combination was significantly higher than that of age, KL-6, D-dimer, or CA19-9 alone (*Z* = 3.248, *p* = 0.001; *Z* = 4.256, *p* < 0.001; *Z* = 4.196, *p* < 0.001; and *Z* = 4.523, *p* < 0.001). The diagnostic efficiencies of the four biomarkers were summarized in Table 2. Taken together, these observations showed that the multivariate models outperformed single biomarkers in diagnosing RA-ILD.

**Table 2:** The predictive power of multiple biomarkers in the diagnosis of patients with RA-ILD vs. RA-non-ILD

	Cut-off	AUC (95%CI)	*P*	Sen (95%CI)	Spe (95%CI)	+ LR (95%CI)	− LR (95%CI)	Youden index	*Z*	*P*′
Age (years)	> 49	0.814 (0.731–0.880)	< 0.001	93.33 (85.10–97.80)	67.95 (56.40–78.10)	2.91 (2.10–4.00)	0.10 (0.04–0.20)	0.613	3.248	0.001
KL-6 (U/mL)	> 373.65	0.749 (0.660–0.824)	< 0.001	61.33 (49.40–72.40)	78.21 (67.40–86.80)	2.81 (1.80–4.40)	0.49 (0.40–0.70)	0.396	4.256	< 0.001
D-dimer (ng/mL)	> 716	0.749 (0.660–0.824)	< 0.001	59.46 (47.40–70.70)	82.19 (71.50–90.20)	3.34 (2.00–5.70)	0.49 (0.40–0.70)	0.417	4.196	< 0.001
CA199 (KU/L)	> 8.01	0.727 (0.637–0.805)	< 0.001	56.52 (44.00–68.40)	77.36 (63.80–87.70)	2.50 (1.50–4.30)	0.56 (0.40–0.80)	0.339	4.523	< 0.001
All		0.928 (0.865–0.968)	< 0.001	83.82 (72.90–91.60)	81.63 (68.00–91.20)	4.56 (2.50–8.30)	0.20 (0.10–0.30)	0.655		

*P* value indicates that the AUC of each indicator has statistical significance. *Z* and *P*′ indicated that the AUC of each indicator had a statistical difference with the AUC of combined detection of the four indicators

*Sen*, sensitivity; *Spe*, specificity; *LR*, likelihood ratio

### Associations between biomarkers and disease activity indicators

**Fig. 6:**
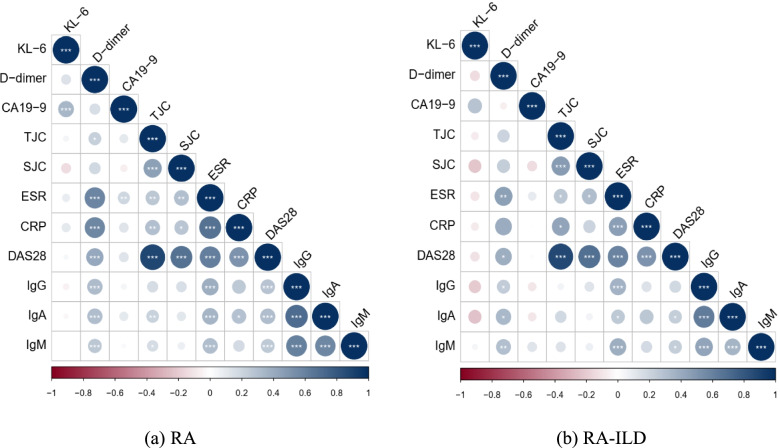
Heatmap of correlation between the biomarkers and disease characteristics. D-dimer was positively associated with disease activity index in patients with RA (**a**) and RA-ILD (**b**), but no correlations between KL-6 and disease activity. * = *p* < 0.05, ** = *p* < 0.001, and *** = *p* < 0.001 by Spearman correlation test

The correlation analysis between biomarkers and disease activity was conducted in RA and RA-ILD patients (Fig. 6). Significant positive correlations were found between D-dimer level and disease activity index in all RA patients, such as ESR (*r* = 0.586, *p* < 0.001), CRP (*r* = 0.574, *p* < 0.001), DAS28 (*r* = 0.414, *p* < 0.001), IgG (*r* = 0.326, *p* < 0.001), IgA (*r* = 0.318, *p* < 0.001), and IgM (*r* = 0.261, *p* < 0.001). The CA19-9 level were weakly correlated with the ESR (*r* = 0.199, *p* = 0.008), but we found no correlations between KL-6 and disease activity indicators (*p* > 0.05), suggesting that KL-6 and CA19-9 might be involved in the pathogenesis of ILD rather than RA. Further analysis proved that there was no obvious correlation between the KL-6 and CA19-9, and any disease activity indicator, in patients with RA-ILD (all *p* > 0.05).

## Discussion

ILD, the most common and serious complication of RA, can occur at any stage of RA. Paradoxically, despite the lung involvement, patients with RA-ILD may remain asymptomatic long-term [3]. Respiratory symptoms (cough, wheezing, or dyspnea) are not obvious in most RA-ILD patients, bringing about challenges to diagnosis, early discovery, and management [21]. With the disease progresses, respiratory failure may develop, leading to poor prognosis and clinical death of patients [22]. The pathogenesis of RA-ILD remains incompletely understood, although genetic, humoral, and environmental factors seem to be involved. Older age, autoantibodies production (anti-CCP and RF), and cigarette smoking may increase the incidence of ILD [23, 24].

We found that the higher frequency of RA-ILD than RA-non-ILD in men, but no significant difference. This may be due to smoking being strongly associated with ILD in males. There was no significant difference in smoking between RA-ILD and RA-ILD groups (21.33% vs 11.54%) in the study, but the odds ratio was 2.079 (Supplementary table 2). Kelly et al. [25] showed the male:female ratio was 1:1.09 in 230 patients with RA-ILD and smoking was associated with ILD in males. In addition, most of the patients with RA-ILD were RF seropositive, older than RA-non-ILD patients. Consistent with our finding, Lee et al. [26] and Kass et al. [27] showed the mean age was significantly higher in the ILD group. The RA-ILD patients had higher levels of disease activity indicators (ESR, CRP, WBC count, and NEUT count), suggesting that ILD might aggravate primary RA. Therefore, it is essential to systematically screen for RA-ILD biomarkers; this permits the management of early-stage of ILD. Over the past decade, several biomarkers diagnostic of RA-ILD have emerged [28, 29]. However, most studies focused on single markers. To the best of our knowledge, this is the first study using a machine learning algorithm to identify multiple biomarkers for RA-ILD, though our data concern a small sample size. Common parameters selected using multiple biostatistical methods are more likely to represent the strongest and true pictures in the data.

We found that the levels of KL-6 and tumor markers (CA19-9, CA242, and CEA) were elevated in RA-ILD patients. Previous studies suggested that RA-ILD patients had significantly higher serum KL-6 and tumor markers than did those without ILD, and that these markers were strongly associated with the severity of ILD [13, 28]. KL-6 is chemotactic for lung fibroblasts and exerts pro-fibrotic and anti-apoptotic effects on these cells [28]. It remains unclear why the levels of tumor markers were elevated, but the results (especially CA199 and CEA) are consistent with observations from patients with CTD-ILD [29, 30]. Wang et al. assessed the levels of various serum tumor markers in a cohort of RA-ILD patients without cancer and found that the CA19-9 level was increased compared to that of RA patients without ILD [12]. CEA has been reported to reflect the proliferation and secretion of epithelial cells [31]. CA19-9 is secreted apically from the bronchial gland, and may induce NEUT maturation; the CA19-9 level correlated positively with NEUT count. Persistent epithelial cell damage and NEUT accumulation in the respiratory tract may explain the high levels of CA19-9 [32].

Furthermore, our results showed that the D-dimer level in the RA-ILD group was higher than that in the RA-non-ILD group. This may reflect the fact that D-dimer (a final product of fibrin degradation) is involved in the acute phase of inflammation [14]. In the acute phase of RA, an elevated D-dimer level may reflect upstream tissue damage caused by inflammatory [33]. We further found that the FIB and FDP levels in the RA-ILD group were significantly higher than in the RA-non-ILD group. In addition, the LDH and HBDH levels were significantly elevated in patients with RA-ILD, providing a new perspective for diagnosing RA-ILD. This may be due to the up-regulation of LDH expression by mammalian target of rapamycin (mTOR) activation on downstream targets, which further leads to the increase of serum HBDH levels [34]. mTOR is a key regulator of cell growth, activation, proliferation, and survival, and is involved in the occurrence and development of both RA and ILD [35, 36].

Subsequently, we used three machine learning algorithms to classify patients with RA-ILD and RA-non-ILD and to assess the importance of various parameters in terms of patient classification. Machine learning models that afford good predictive accuracy can be used to generate reliable biomarkers [17]. We augmented the model strength and stability by running the training iterations tenfold cross-validation and constructing 10 different data segmentation models. Such tenfold cross-validation simulates the more standardized diagnostic test and affords better classification [37]. Interestingly, all three approaches delivered highly consistent results. The best AUCs of the LASSO, RF, and PLS were all 0.95, suggesting that the identified markers robustly enhance current disease classification. Using the Lasso, RF, and PLS, RA patients are likely to be correctly classified as ILD or non-ILD. Our methods are the first to identify serum features associated with RA-ILD. However, machine learning does not replace traditional analytical analyses, rather further assisting clinical diagnosis by enhancing existing methods.

Importantly, four indicators, age, KL-6, D-dimer, and CA19-9, were identified as the most valuable biomarkers by the three machine learning algorithms and univariate analysis; and the four biomarkers might be involved in the occurrence and development of ILD. Notably, the ROC curve for the combination of age, KL-6, D-dimer, and CA19-9 exhibited an AUC of 0.928, a sensitivity of 83.82%, and a specificity of 81.63%. We further explored the correlations between biomarkers and ILD. Remarkably, we found no correction between the KL-6 or CA19-9 level and disease activity, indicating that KL-6 and CA19-9 may be independent predictors independent of disease activity and might be involved in the pathogenesis of the ILD rather than RA. Compared to the other biomarkers, KL-6 has the superior diagnostic value.

Last but not least, the diagnosis of ILD usually depends on HRCT, PFT, and lung ultrasound (LUS). HRCT can identify even subtle ILD changes and monitor existing diseases. However, radiation exposure and high cost restrict its use for screening and monitoring purposes [9]. PFT, especially forced vital capacity and diffusing capacity for carbon monoxide, could help guide management strategies. However, its role in screening for early asymptomatic ILD is controversial due to low sensitivity and poor repeatablility [38]. Over the past two decades, LUS has developed into a promising tool for assessing lung parenchymal disease by detecting and quantifying the number of B lines. However, adequate theoretical and practical training are prerequisites for LUS use. In addition, accurate results require more scanning sites and more time [39]. At first glance, the combination described in this study was based on the measurement of four different blood parameters, which may raise feasibility issues. However, the quantitative measurements of KL-6, D-dimer, and tumor markers in the blood can be performed easily and rapidly in most laboratories. In addition, the inherent characteristics of biomarker, including that it is non-ionizing, non-invasive, at low cost, repeatable, and easily accessible, make the combination possible initial screening tool of RA-ILD and aid clinicians to determine if ILD is present in RA patients [40]. Although the model is logical and easy to use, it still has some shortcomings. In the selection of biomarkers and the development of models, a hold out test set, or an external validation cohort should be employed to validate our findings, which can greatly improve the rigor and accuracy of the study, however, the small sample size limited the execution in this study. Therefore, prospective studies in larger cohorts need to be performed to verify the predictive value of the models.

## Conclusion

In conclusion, we used novel tools to identify biomarkers associated with ILD in an RA cohort. Integration of traditional biostatistical methods with emerging machine learning algorithms yielded simple a model predicting RA-ILD, which may provide a new idea for future studies on the diagnosis of ILD and could also be generalized to predict the involvement of other organs.

## Supplementary Information

**Additional file 1:** **Supplemental Table 1 **Performance ofmultiple machine learning classifiers to predict RA-ILD by 10-foldcross-validation. **Supplemental Table 2 **Comparisons between smoking and RA-ILD.

## Data Availability

All data generated or analyzed during this study are available from the corresponding author on reasonable request.

## Abbreviations

RA: Rheumatoid arthritis
ILD: Interstitial lung disease
HRCT: High-resolution computed tomography
KL-6: Krebs von den Lungen-6
CTD-ILD: Connective tissue disease-related interstitial lung disease
CA: Carbohydrate antigen
ACR: American College of Rheumatology
TJC: Tender joint count
SJC: Swollen joint count
DAS28: Disease activity index 28
WBC: White blood count
RBC: Red blood cells
Hb: Hemoglobin
PLT: Platelets
LYMPH: Lymphocytes
NEUT: Neutrophils
ESR: Erythrocyte sedimentation rate
CRP: C-reactive protein
ALT: Alanine transaminase
AST: Aspartate aminotransferase
TP: Total protein
ALB: Albumin
GLO: Globulin
LDH: Lactate dehydrogenase
HBDH: Lactate dehydrogenase
Ig: Immune globulin
RF: Rheumatoid factor
ANA: Anti-nuclear antibodies
APF: Anti-perinuclear factor
AKA: Anti-keratin antibodies
MCV: Anti-mutated citrulline vimentin
CCP: Anti-cyclic citrullinated peptide antibody
FDP: Fibrinogen degradation products
FIB: Fibrinogen
NSE: Neuron-specific enolase
CEA: Carcinoembryonic antigen
SCC: Squamous cell carcinoma antigen
AFP: Alpha fetoprotein
LASSO: Least absolute shrinkage and selection operator
RF: Random forest
PLS: Partial least square
ROC: Receiver operating characteristic
AUC: Area under curve
Sen: Sensitivity
Spe: Specificity
+ LR: Positive likelihood ratio
–LR: Negative likelihood ratio
mTOR: Mammalian target of rapamycin
