# Supplementary material for: Identification of biomarkers by machine learning classifiers to assist diagnose rheumatoid arthritis-associated interstitial lung disease
Source: Arthritis Res Ther. 2022 May 19;24:115. doi: 10.1186/s13075-022-02800-2 (PMC9118651; doi:10.1186/s13075-022-02800-2)
Supplement: Supplementary file 1 — Additional file 1: Supplemental Table 1 Performance ofmultiple machine learning classifiers to predict RA-ILD by 10-foldcross-validation. Supplemental Table 2 Comparisons between smoking and RA-ILD. [file 13075_2022_2800_MOESM1_ESM.docx]

**Supplemental Table 1** Performance of multiple machine learning classifiers to predict RA-ILD by 10-fold cross-validation

|  | LASSO-AUC | RF-AUC | PLS-AUC |
| --- | --- | --- | --- |
| Model 1 | 0.92 | 0.85 | 0.90 |
| Model 2 | 0.84 | 0.87 | 0.86 |
| Model 3 | 0.84 | 0.90 | 0.86 |
| Model 4 | 0.91 | 0.87 | 0.82 |
| Model 5 | 0.93 | 0.91 | 0.85 |
| Model 6 | 0.94 | 0.87 | 0.85 |
| Model 7 | 0.93 | **0.95** | **0.95** |
| Model 8 | 0.84 | 0.86 | 0.81 |
| Model 9 | 0.93 | 0.91 | 0.87 |
| Model 10 | **0.95** | 0.93 | 0.85 |
| Mean ± SD | 0.903 ± 0.04 | 0.892 ± 0.03 | 0.862 ± 0.04 |

AUC, area under the curve; SD, standard deviation.

**Supplemental Table 2** Comparisons between smoking and RA-ILD

|  | Smoking | | Total | OR |
| --- | --- | --- | --- | --- |
|  | Yes | No |  |  |
| RA-ILD | 16 | 59 | 75 | 2.079 |
| RA-non-ILD | 9 | 69 | 78 |  |
| Total | 25 | 128 | 153 |  |

OR:odds ratio
